# Uncovering Spatial Variation in Acoustic Environments Using Sound Mapping

**DOI:** 10.1371/journal.pone.0159883

**Published:** 2016-07-28

**Authors:** Jacob R. Job, Kyle Myers, Koorosh Naghshineh, Sharon A. Gill

**Affiliations:** 1 Department of Biological Sciences, Western Michigan University, Kalamazoo, MI, United States of America; 2 Department of Mechanical and Aerospace Engineering, Western Michigan University, Kalamazoo, MI, United States of America; University of Windsor, CANADA

## Abstract

Animals select and use habitats based on environmental features relevant to their ecology and behavior. For animals that use acoustic communication, the sound environment itself may be a critical feature, yet acoustic characteristics are not commonly measured when describing habitats and as a result, how habitats vary acoustically over space and time is poorly known. Such considerations are timely, given worldwide increases in anthropogenic noise combined with rapidly accumulating evidence that noise hampers the ability of animals to detect and interpret natural sounds. Here, we used microphone arrays to record the sound environment in three terrestrial habitats (forest, prairie, and urban) under ambient conditions and during experimental noise introductions. We mapped sound pressure levels (SPLs) over spatial scales relevant to diverse taxa to explore spatial variation in acoustic habitats and to evaluate the number of microphones needed within arrays to capture this variation under both ambient and noisy conditions. Even at small spatial scales and over relatively short time spans, SPLs varied considerably, especially in forest and urban habitats, suggesting that quantifying and mapping acoustic features could improve habitat descriptions. Subset maps based on input from 4, 8, 12 and 16 microphones differed slightly (< 2 dBA/pixel) from those based on full arrays of 24 microphones under ambient conditions across habitats. Map differences were more pronounced with noise introductions, particularly in forests; maps made from only 4-microphones differed more (> 4 dBA/pixel) from full maps than the remaining subset maps, but maps with input from eight microphones resulted in smaller differences. Thus, acoustic environments varied over small spatial scales and variation could be mapped with input from 4–8 microphones. Mapping sound in different environments will improve understanding of acoustic environments and allow us to explore the influence of spatial variation in sound on animal ecology and behavior.

## Introduction

A major goal of animal ecology is to understand the features of the environment that influence the distribution of animals over space and time. To achieve this goal, researchers explore biotic and abiotic drivers relevant to the species under study, yet among potential drivers, the acoustic environment is rarely considered [1]. Sound will, of course, be highly relevant to taxa that rely on acoustic communication to achieve fitness, but given the pace and extent of land transformation [2], it is becoming increasingly evident that many taxa may be affected by changes to the acoustic environment. This is because a widespread outcome of land transformation is increased levels of anthropogenic noise [3], which mainly consists of high amplitude, low frequency sound emitted by diverse sources, including vehicles, urban infrastructure and industrial machinery [4,5]. Anthropogenic noise (hereafter noise) has widespread effects on animals, not only by disrupting vocal communication [6], but also through effects on vibrational communication (e.g., [7]), foraging (e.g., [8]), predator detection (e.g., [9]), stress (e.g., [10]), and reproductive success (e.g., [11]). Given these negative effects, organisms might be expected to avoid habitats with high noise levels [12–15]. If they cannot avoid noisy habitats, organisms might use space within their home ranges or territories as a function of heterogeneity in noise over space and time.

To assess whether noise influences habitat selection and space use, ecologists need to incorporate acoustic features into habitat descriptions [1] at multiple spatial scales [16]. While it seems quite obvious that noise and sound more generally varies spatially and temporally, the reality is that we rarely measure sound in ways that reflect its dynamic nature [5,17]. One promising avenue is the adoption of noise maps. Noise maps provide graphical representations of sound pressure levels (SPLs) over defined spatial and temporal scales in cities [18], and are modeled based on inputs from SPL monitoring, weather, human activity, and environmental structure [19,20]. Recently, noise mapping has been used to explore SPLs on large spatial scales that include natural areas in both marine (e.g., [21,22]) and terrestrial systems (e.g., [23,24]). These studies show that sound varies considerably over large spatial scales and have identified landscape-level drivers of spatial variation in sound, including land cover, precipitation, and modes of transportation.

We propose that advances in animal ecology can be achieved by mapping sound at smaller spatial scales. Critical understanding of animal responses to noise comes from individual- and population-level studies (reviewed in [25]), but existing methodology for noise mapping does not provide details about variation in sound pressure levels (SPLs) at scales relevant to individuals and populations. Modeled noise maps may have limited input from sound recordings [26], resulting in maps that mismatch modeled and actual readings of noise levels [19] and poorly depict noise patterns over space and time (e.g., [27]). Noise maps may model sound propagation based on SPLs measured at a single source, yet in wildlife habitat with large numbers of sound sources, such modeling may be unrealistic [24]. Further, noise maps typically show time-integrated measurements of SPLs (e.g. daytime continuous averages), but such averages obscure SPLs over hours, minutes and seconds, that is, over timescales relevant to individual behavioral decisions, including adjustments to noise (e.g., [5,28,29]). Therefore, to understand animal responses to spatial variation in noise as well as sound more generally, an approach is needed that provides fine-scale resolution of sound variation over space and time.

Here, we adapt the idea of noise mapping for use at small spatial scales relevant to the ecology of diverse animal taxa. Given their widespread and successful use in studies of animal localization, movements, and communication (e.g., [30,31]), we used microphone arrays to record the sound environment of three terrestrial habitats. Microphone arrays consist of multiple microphones set to record simultaneously over a defined area and defined time period and thus our aim was to map actual readings of sound pressure levels (SPLs) to explore the extent to which sound varies over small spatial scales. We assessed the minimum number of microphones under ambient conditions and noise introductions that resulted in maps similar to those created using arrays with greater coverage, which allowed us to make recommendations for implementing sound mapping for future studies. We propose sound mapping using input from microphone arrays as a tractable approach to advance our understanding of how acoustic environments vary spatially, which when combined with individual- and population-level studies will add a needed dimension to research on animal responses to anthropogenic noise.

## Methods

We set up microphone arrays during July—August 2013, on days without rain and wind (wind speed < 7 m/s) in Kalamazoo County, MI, U.S.A. We ran trials at five separate locations in each of three environments for a total of 15 trials. The urban site was the campus of Western Michigan University (42.283 N, 85.614 W), prairie sites consisted of short-grass prairies at the Kalamazoo Nature Center (42.362 N, 85.583 W), and mature oak and hickory forest at Asylum Lake Preserve (42.264 N, 85.641 W) and Fort Custer Recreation Area (42.325 N, 85.352 W) acted as our forest sites. We placed arrays in the urban site in relatively open areas, with lawn and sidewalks plus scattered trees and shrubs. No man-made structures occurred within 75 m of array locations in prairie and forest sites. We set arrays in areas that were relatively level, but we estimated elevation gains of 5–10 m at two forest sites and two prairie sites.

### Ethics Statement

Permission to conduct research was granted by Kalamazoo Nature Center, the Asylum Lake Policy and Management Council (Asylum Lake Preserve), and the Michigan Department of Natural Resources, Parks and Recreation Division (Fort Custer Recreation Area). No specific permission was required to work on the campus of Western Michigan University.

### Recording units and microphone arrays

To record sound within arrays, we deployed 14 programmable Song Meter 2 (SM2s) + GPS recording units (Wildlife Acoustics, Inc., Maynard, MA, USA). Each recorder has ports for two omnidirectional microphones giving us 28 microphones to position within arrays. We used SM2s outfitted with Garmin GPS units to ensure clock synchronization of 1 ms between recorders within arrays. We set sampling rate at 22050 Hz and gain at +24 dB and saved recordings as 16-bit.wav files on 32GB SD cards housed within units (see [31] for discussion of SM2s).

**Fig 1:**
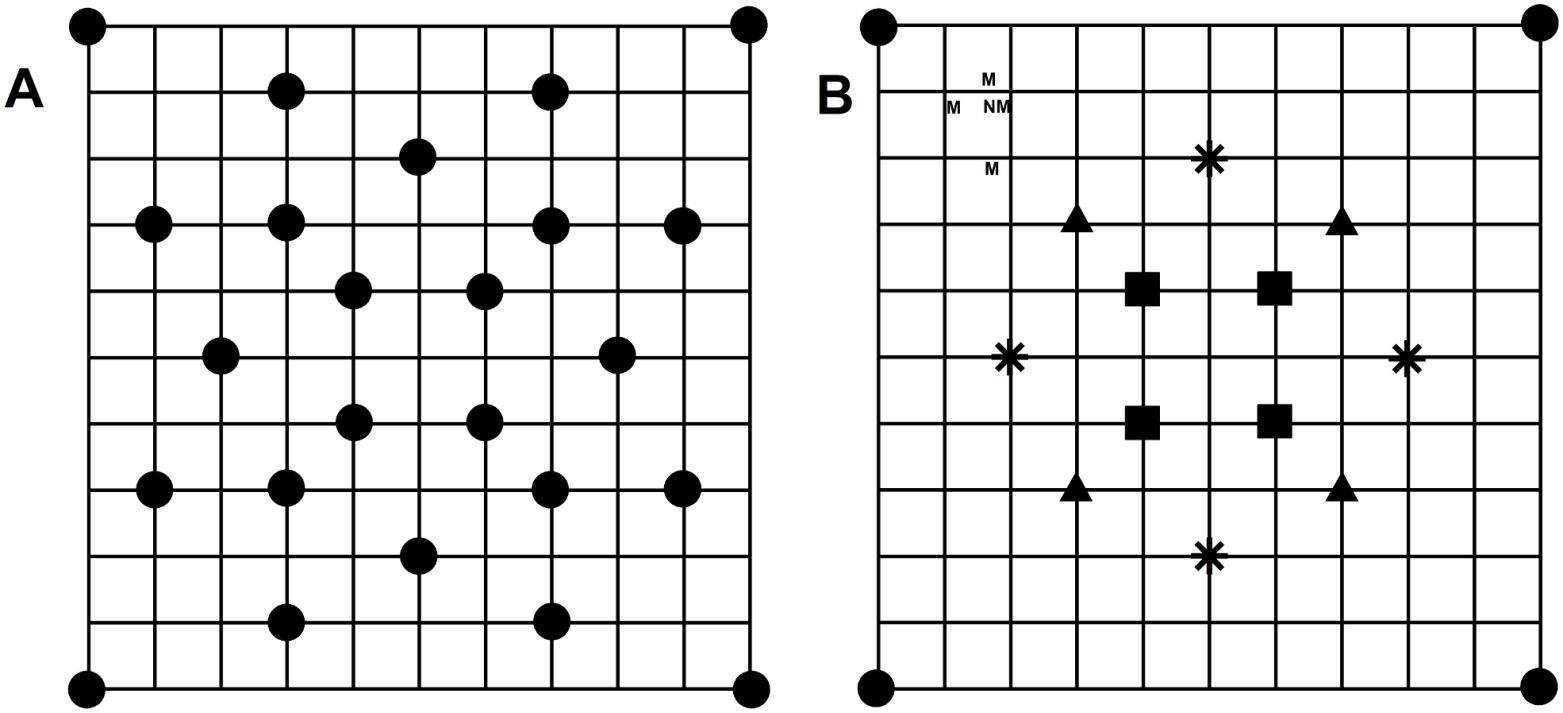
Layouts of microphone arrays used to record the sound environment. (A) Layout of full 24-microphone array used to record the ambient environment and (B) nested microphone subset arrays (● = 4, ■ = 8, ▲ = 12, and * = 16) used during noise introductions. As examples, the 8-microphone subset array included four central microphones (■) and four corner microphones (●), and the 12-microphone array included these 8 microphones plus four more (▲). ‘N’ represents a hypothetical location for noise introduction and each ‘M’ represents one of four additional microphones surrounding the noise source. The overall spatial extent is 60 m x 60 m for the entire plot and 6 m x 6 m for internal squares.

**Fig 2:**
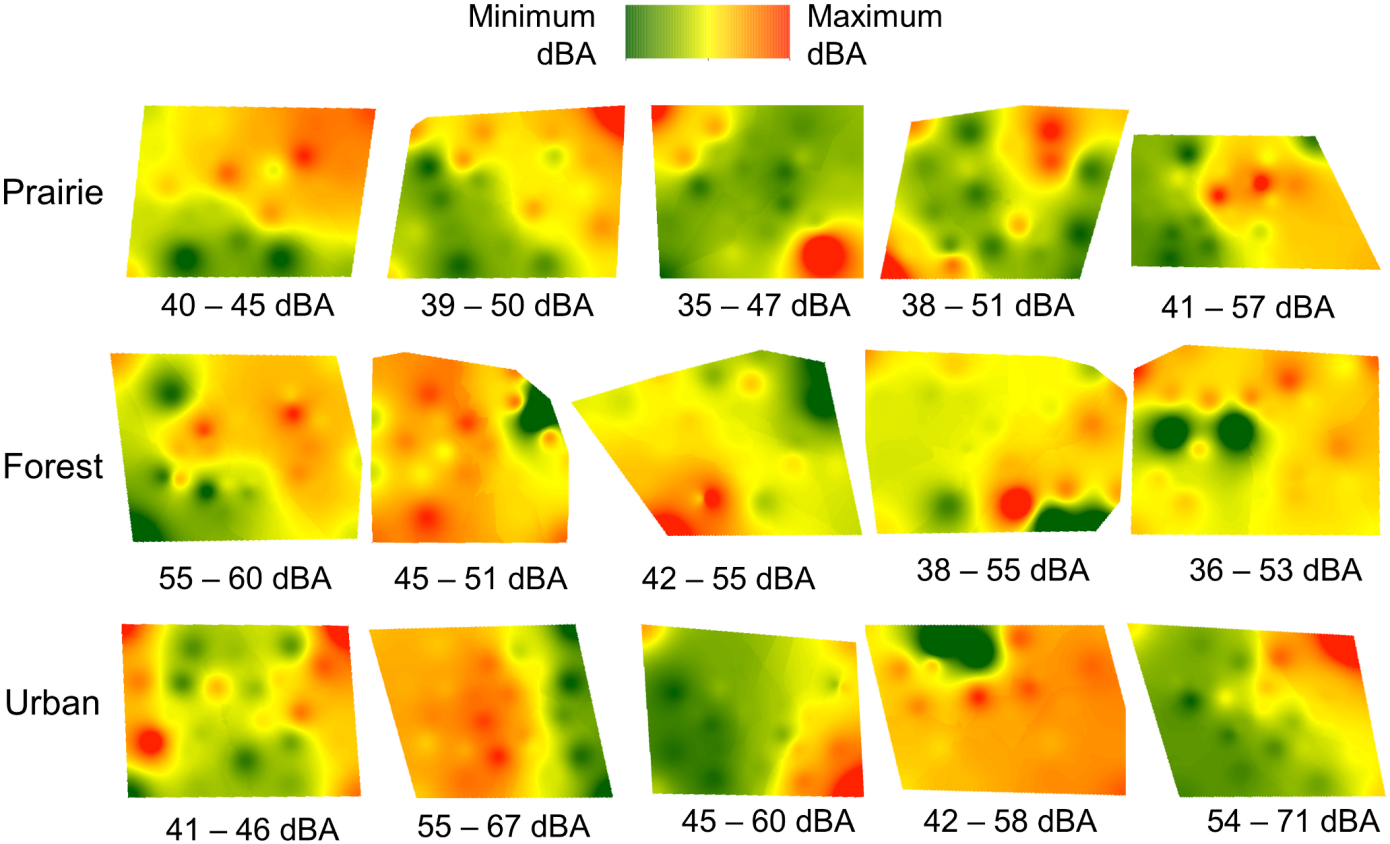
Sound maps of ambient conditions across each of five sites in each of three habitats. Maps were generated using full arrays of 24 microphones and show overall sound pressure levels (SPL, dBA). Note that the color scale is the same for all maps, but the actual SPLs reflected by the scale differ across maps and are indicated by the range of values below each map; we presented each with a unique scale to best visualize spatial variation in SPLs within a site. Maps within each habitat are ordered from the smallest to the largest range of SPLs within a given habitat type.

We set up 24-microphone arrays over a 60 m x 60 m area, using the layout shown in Fig 1A (errors in array deployment and GPS locations contributed to variation in shape of maps in Fig 2; area of maps was consistent across arrays, mean ± SE = 0.36 ± 0.01 ha). We taped microphones to the top of 2 m poles and used 3–50 m cables to connect each microphone to its SM2 recorder. By using cables, we took advantage of the two microphone ports per recorder to place microphones in locations that improved coverage of the plot. We selected a height of 2 m for microphones to minimize effects of ground attenuation, particularly in the open urban site [32]. We randomly selected two locations, one within the array (> 6 m from the outside edge of the array) and the other on its edge (< 6 m from the boundary), as sites for noise introductions. At the location for the first trial, we placed a custom-built speaker (see below) and positioned four additional microphones 2, 4, 6, and 8 m and at 90° increments from the speaker and more than 2 m from other microphones within arrays. For playback locations at array edges, extra microphones were placed within array boundaries. After microphones were set, we recorded their locations and the speaker with a Garmin GPSmap 60CSx unit placed at each microphone for 5 min (accuracy: 2.5 m at prairie and urban sites; 3.5 m at forest sites). After the first noise introduction, we moved the speaker and additional microphones to the second location, took GPS locations, and ran the second noise trial.

### Sound recording within arrays

We recorded sound fields for 5 min under ambient conditions, which we defined as the sound environment produced by natural sources, such as wildlife and rustling of leaves, and human activities [33], and during noise introduction experiments performed within arrays and at their edges (order of playback location was randomized across trials). We used noise experiments to assess whether additional microphones around noise sources were needed to capture steep noise gradients (e.g., [27]). We used 24-microphone arrays (Fig 1A) to record ambient conditions. During the two experimental periods, we added four microphones (28 microphones total) as described above and randomly selected one of five recordings of traffic noise to play within the array (recorded 1 m from high-traffic intersections in Kalamazoo, MI, between 0700–0800; see S1 Fig for spectrograms and power spectra for 1-min samples of each recording). We used an SPL-8010 meter (American Recorder Technologies, A-weighting, fast response) to measure playback volume at 1 m from the speaker and broadcast noise such that maximum SPL during playback was 90 dBA. The speaker was a custom-built plywood box outfitted with a 12 V battery, 100 W/channel Sound Ordnance M4100 amplifier, and five 16.5 cm 250 W Pioneer TS-G1644R speakers (frequency response 31 Hz to 29 kHz at -20 dB) arranged to produce omnidirectional noise (one speaker on each side and the top of the box).

### Microphone calibrations and data extraction

From recordings, we extracted overall sound pressure levels (SPLs). SPLs can be extracted from.wav files if microphone-recorder systems have been calibrated to known SPLs at known gain settings [5]. We labeled all SM2s, microphones, and ports to ensure that SM2-microphone-port combinations used in the field were the same as those calibrated in the lab. We calibrated SM2s and microphones inside an anechoic chamber using a Larson Davis CAL 200 Precision Acoustic Calibrator, which generates a 1 kHz tone at 94 dB as a calibration signal. The outcome of this process was a single dB–Volts to dB SPL correction factor (i.e., a calibration factor) for each SM2-microphone-port combination. We used calibration factors in a custom script in MATLAB Version 7.9 (Natick, MA, USA: MathWorks Inc., 2009) to extract overall A-weighted SPLs (dBA) averaged over 30-s intervals throughout the 5-min recordings. The selection of 30-s intervals was arbitrary, but selections could be timed to coincide with focal animal spot sampling, with changes in behavior of focal animals, or based on a priori knowledge of temporal fluctuations of the sound environment (e.g. onset of rush hour). A-weighting approximates the hearing threshold of passerines and humans [34], but other weightings, including flat- and species specific-weightings, could be used instead (see [5]).

### Sound mapping

We combined overall SPLs for each microphone with corresponding GPS coordinates of microphone locations in spreadsheets and converted them to shapefiles of microphone locations using ArcGIS ArcCatalog (V10.2, Environmental Systems Resource Institute (ESRI), Redlands, CA, USA). We used inverse distance weighting (IDW) interpolation in ArcGIS ArcMap (V10.2, ESRI, Redlands, CA, USA) to create interpolated raster sound maps from SPLs. IDW interpolation includes a distance decay power parameter, which we set to 2 for all maps to mimic natural sound decay in a free-field environment as modeled by the inverse square law [35]. SPLs from all locations were used for the sound surface estimate in analysis of 4, 8 and 12 microphone configurations. For configurations of greater than 12 microphones, the nearest 12 microphones were used as inputs for IDW, which reduced the search neighborhood as more data became available.

We created subset maps by sequentially excluding microphones and their SPLs during the mapping procedure to evaluate the effect of decreasing the number of microphones used to generate sound maps across sound conditions and habitats. For ambient conditions, we created 4, 8-, 12-, and 16-microphone subset arrays (Fig 1B). For noise introductions, we created two groups of subset arrays. One group consisted of the 4-, 8-, 12-, and 16-microphone subsets and the second also included the four additional microphones around the speaker resulting in 8-, 12-, 16- and 20-microphone subset arrays (Fig 1B). To distinguish between the groups, we used the convention n+4 to indicate maps with additional microphones around the noise source (e.g. 4+4 array).

### Map analysis

To evaluate whether the number of microphones affected mapping of the sound environment, we calculated SPL differences (dBA) between maps generated using full versus subset arrays. We assumed full array maps most accurately captured the sound environment; therefore, we considered them the reference against which we tested subset maps. We compared full 24-microphone array maps against 4-, 8-, 12-, and 16-microphone subset arrays for both ambient and noise conditions (i.e., without microphones around the speaker), as well as 24+4-microphone arrays against 4+4, 8+4, 12+4, and 16+4 microphone arrays (i.e., with the four additional microphones) for noise introductions. Within the Spatial Analyst extension in ArcGIS, we used the Map Algebra toolset to subtract subset maps from their corresponding full array maps, producing difference maps in which each pixel represents the difference in SPLs at a given point between full and subset arrays maps. We selected a pixel size of 0.47 m x 0.47 m (0.22 m^2^), which is less than the minimum distance between microphones (2 m) in arrays. Small pixel sizes increase resolution and spatial accuracy, which is important when dealing with heterogeneous variables. We calculated mean pixel difference (dBA) for each difference map. Differences between maps could be positive or negative; we used absolute values in analyses to examine the magnitude of differences between full and subset array maps. To assess if subset maps consistently under- or overestimated full map values, we summarized the number of subset maps that under- or overestimated the full maps and calculated the mean difference for these maps (S1 Table).

### Statistical analyses

To describe the sound environment across sites and sound conditions, we compared overall SPLs among habitats under ambient and noise conditions using two-way ANOVAs and Tukey HSD post-hoc tests and examined differences in overall SPLs between noise treatments across habitats using t-tests. To analyze the effect of the number of microphones on sound maps, we first analyzed whether subset maps differed from full maps using one-sample t-tests, testing if mean pixel differences were different than zero. We then explored differences among the subset maps themselves by analyzing mean pixel differences associated with subset maps under ambient conditions and noise introductions within and on edges of arrays. For ambient conditions, we performed a two-way ANOVA with interactions and microphone subset and habitat types as factors. For noise introductions, we performed a four-way ANOVA with interactions, which, in addition to microphone subset and habitat, included noise location (within versus edge of arrays) and the use of additional microphones (yes or no). We examined fully crossed initial models using Levene’s test to examine equality of variance and plots of residual vs. fitted values to examine normality. The dependent variable in both models was log transformed prior to analysis. We used R v. 3.0.2. [36] for all analyses.

We also considered whether differences were likely to be detectable by organisms by interpreting results in terms of a 3-dBA difference threshold. Results may be statistical significant, but differences between maps of less than 3 dBA may not detectable or meaningful to some species. Humans and birds appear to be unable to detect differences in SPLs of less than 3 dBA [37,38], but frogs [39] and insects [40] are capable of discriminating smaller intensity differences; hence, we present statistical results within the context of threshold differences.

## Results

### Sound maps of ambient conditions

Sound intensity under ambient conditions differed among the three habitats. Mean overall SPLs were significantly lower within prairie arrays versus urban arrays; SPLs from forest arrays were intermediate and did not differ significantly from urban or prairie arrays (Table 1). Sound maps of ambient conditions revealed the extent to which overall SPLs varied spatially across the three habitats (Fig 2). Each map represents one randomly selected time point within the five-minute recording periods. The maps reveal considerable spatial detail in the sound environment, as no sites were homogeneous with respect to overall SPLs. In these examples, maximum and minimum SPLs within a site differed by up to 17 dBA, although four sites from each of the three habitats showed relatively small differences in SPLs (5–6 dBA) over space. Differences between maximum and minimum SPLs were similar among habitats (F_2,12_ = 0.21, P = 0.8; S2 Fig).

**Table 1:** Sound pressure levels (SPL, dBA) under ambient conditions and with noise introductions in three habitats. Data are presented as means ± standard errors and ranges**.** Results from one-way ANOVAs compare SPLs among habitats under each sound condition.

			Location of noise introduction			
Habitat	Ambient conditions		Within array		On edge of array	
Prairie	43.7 ± 0.5 *	29.1–61.6	67.8 ± 0.3 *	45.5–80.1	67.3 ± 0.5 *	39.2–81.6
Forest	50.4 ± 0.5	34.6–59.7	76.9 ± 0.6 *†	53.5–94.0	77.4 ± 0.5 *†	40.8–93.1
Urban	60.6 ± 1.4 *	36.8–73.3	70.5 ± 0.5 †	46.3–85.6	70.5 ± 0.5 †	43.4–89.4
**F**_**(d.f.)**_**; P**	7.96_(2,12)_; < 0.01		7.71_(2,12)_; < 0.01		7.61_(2,12)_; < 0.01	

SPLs that share the same symbols (either * or †) differed significantly (P < 0.05; post-hoc Tukey HSD tests).

SPLs mapped every 30 s over the 5-min recording periods reveal temporal variation in sound (animation of maps from one randomly selected site from each habitat are available online in S1 Movie). Minimum and maximum SPLs at these sites differed by 15–21 dBA over 5 min, but locations of quieter and noisier areas within these sites were somewhat consistent over time. For example, in the prairie sound map sequence, a high SPL area occurs in the upper right hand corner of the map at 9 of 10 time points (generated by an indigo bunting, *Passerina cyanea*, singing near the microphone), with two additional high SPL areas emerging at 2 other times. In the forest sound map sequence, a low SPL area occurs in the bottom right hand corner of the map throughout the sequence, with high noise areas appearing in several areas over time. The urban sound map sequence was more consistent, with more subtle changes occurring over time in the location of mid-range SPLs.

### Are full arrays needed under ambient conditions?

**Fig 3:**
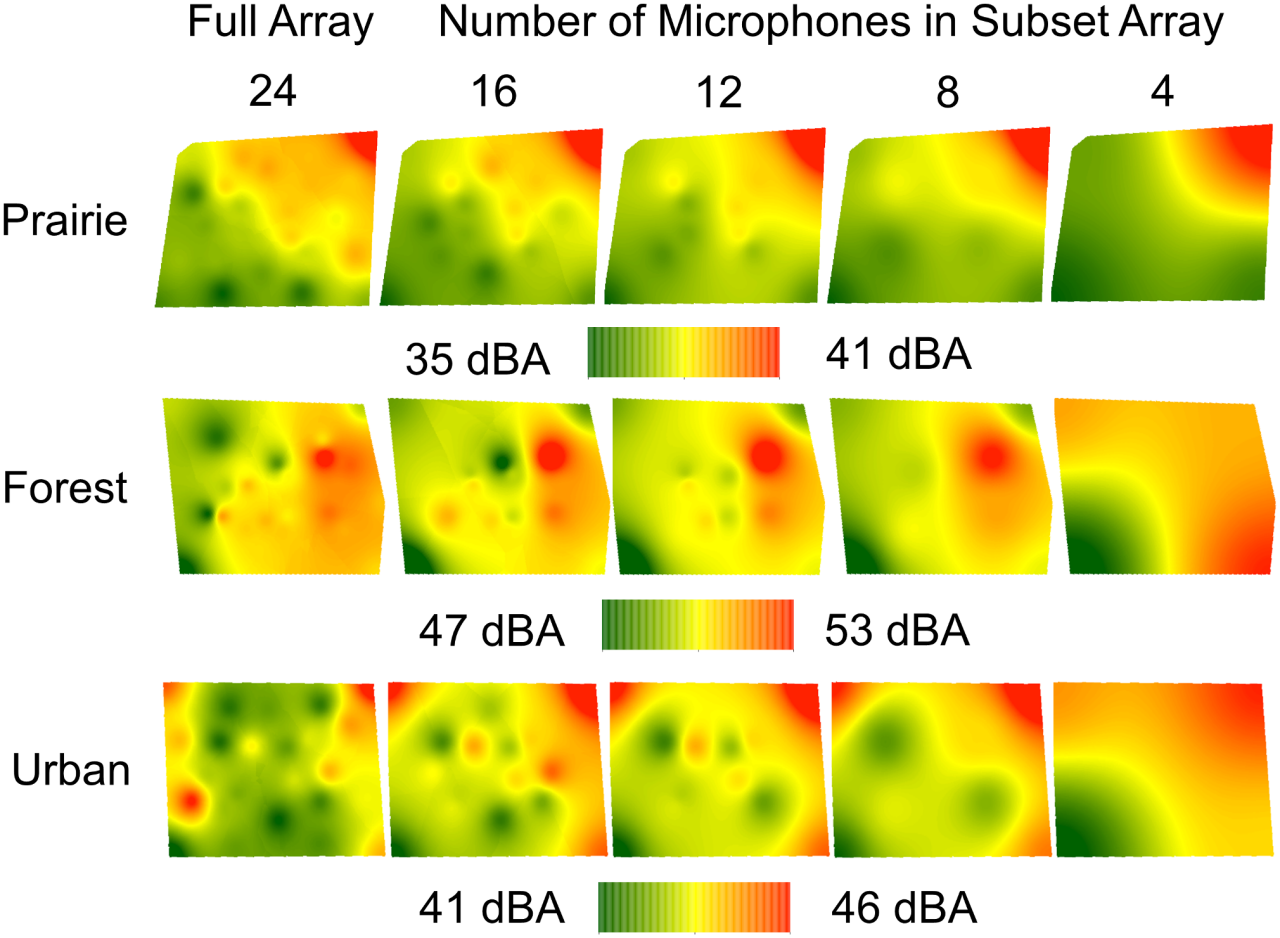
Full and subset sound maps of ambient conditions in three habitats. Full maps were generated using 24 microphones, with subset maps generated by sequentially dropping groups of microphones from full arrays. Each map illustrates the same randomly selected time point within a habitat. Note that the range of SPLs mapped is similar (5–6 dBA), but that the absolute values of SPLs differ among maps for the three habitats.

**Fig 4:**
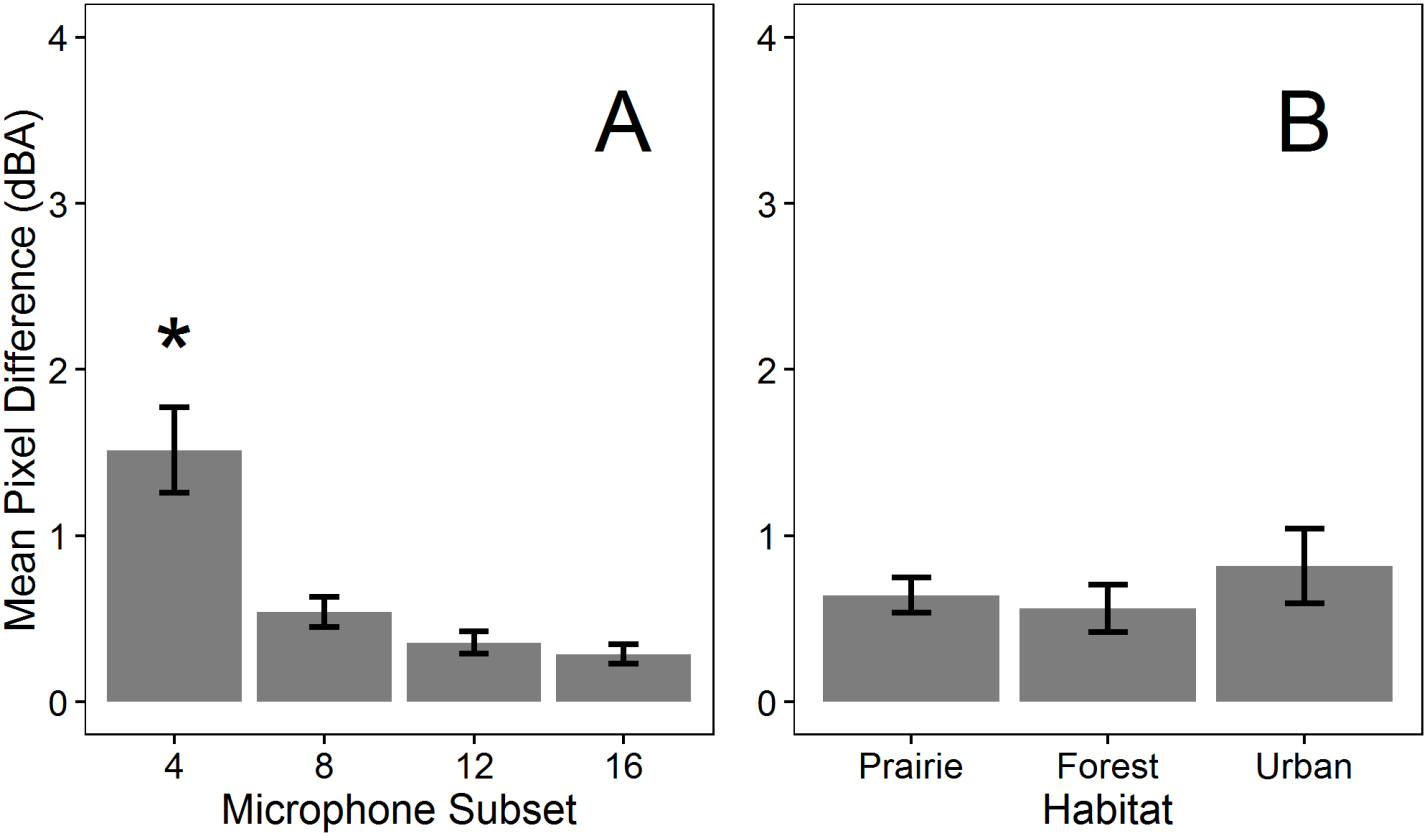
Map differences of ambient conditions varied with microphone number (A) but not across habitats (B). Mean pixel difference is the average difference in overall sound pressure levels (dBA) calculated by subtracting a subset map from the corresponding full array map and averaging overall all pixels. The asterisk denotes a significant difference as tested using Tukey HSD (P < 0.05). Error bars represent standard error.

Examples of sound maps of ambient conditions generated using full and subset arrays across habitats are shown in Fig 3. As the number of microphones used to generate maps decreased, the detailed spatial patterns of SPLs visible in full maps became obscured and only broad gradients from high to low SPLs were visible in 4-microphone maps. Although this change is visible across maps from all habitats, the loss of detail appears greater for forest and urban maps, in which the spatial extent of the gradient visible in the 4-microphone array differs considerably from the spatial variation in SPLs with 8 or more microphones. However, the overall ranges of SPLs in forest and urban maps are narrow (5 and 6 dBA) and in areas within the 4-microphone maps where detailed spatial pattern appears lost (e.g. the upper left corner of the forest map), the actual differences between maps are < 3 dBA. Across habitats, mean pixel differences between full and subset maps were significantly different from zero (t > 4.89, P < 0.001; S2 Table), but for 8-, 12- and 16-microphone subset maps, mean differences (Fig 4A) and the value of the upper confidence interval were both < 1 dBA (S2 Table). Four-microphone maps differed more from full maps than the 8-, 12-, or 16-microphone maps (F_3,48_ = 11.84, P < 0.001, Tukey HSD P < 0.05; Fig 4A), but mean differences between 4-microphone maps and full maps and between 4-microphone maps and the other subset maps were < 2 dBA. Habitat did not influence differences between subset and full maps (F_2,48_ = 1.06, P > 0.3, Fig 4B) and no interaction existed between microphone number and habitat (F_6,48_ = 1.16, P > 0.3).

### Sound maps of noise introductions

**Fig 5:**
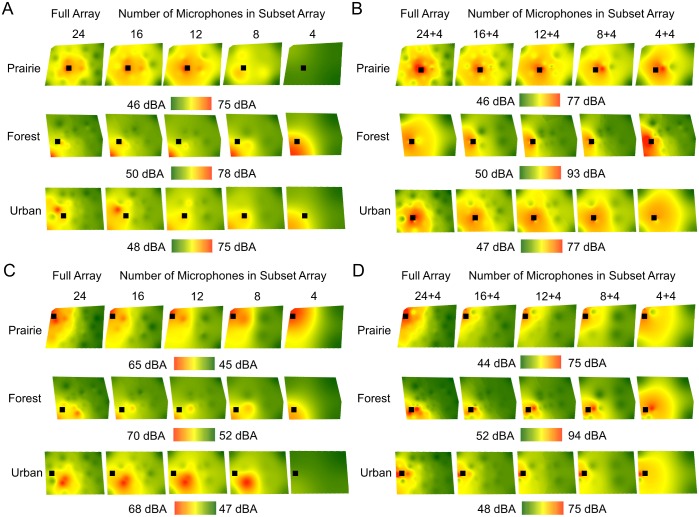
Examples of sound maps illustrating overall SPLs (dBA) during noise introductions (■) in three habitats. Maps show noise introduction within arrays without (A) and with (B) additional microphones around noise source, and noise introduction at the edge of arrays without (C) and with (D) the additional microphones. Within habitat, maps in (A) and (B) illustrate the same randomly selected locations and time points, as do (C) and (D). Arrays are the same as those shown in Fig 3. Note that the ranges and values of SPLs differ among maps.

Unsurprisingly, noise introductions increased overall SPLs compared with ambient conditions when noise was introduced within (t_(14)_ = -10.58, P < 0.001) or at the edge of arrays (t_(14)_ = -12.31, P < 0.001). The location of noise introduction, however, did not affect mean SPLs (t_(14)_ = -0.86, P = 0.40). A range of SPLs observed during noise introductions was observed, indicating that although playbacks increased SPLs overall, they did not homogenize SPLs across the arrays. Mean SPLs during noise introductions were similar in prairie and urban arrays, but significantly higher within forest arrays (Table 1). Differences between minimum and maximum SPLs in forests were greater than in prairie and urban arrays when noise was introduced within arrays (F_2,12_ = 9.41, P = 0.004; S2 Fig). When on the edge of arrays, SPL gradients were steeper in forest than urban arrays only (F_2,12_ = 4.21, P = 0.041; S2 Fig). Sound maps of noise introductions generated using full arrays (24 microphones) and full+additional arrays (24+4 microphones around the noise source) are shown in Fig 5. In these maps, SPLs varied within a given map by up to 43 dBA, with larger ranges in SPLs for the maps created using additional microphones around the noise source (27–43 dBA) than maps without them (21–29 dBA).

### Are full arrays needed when noise is introduced?

As with ambient conditions, the detailed patterns of SPL variation over space became increasingly masked as the number of microphones providing input to maps decreased. Moreover, without additional microphones around the speaker (Fig 5A and 5C), the locations of noise introductions became less clear. For examples, see the 4-microphone map in prairie (Fig 5A) compared with the 4+4 array (Fig 5B), and the 4-microphone map in urban (Fig 5C) compared with the 4+4 array (Fig 5D); in 4-microphone maps, noise introductions are not apparent and maps show little spatial variation in SPLs. Maps created using input from microphones surrounding the noise source (Fig 5B and 5D) showed similar spatial patterns of SPLs even as the number of microphones was reduced. The spatial extent of moderate to high SPLs appeared larger in the 4+4 maps than in the remaining maps, particularly when noise introductions occurred at the edge of arrays (Fig 5B and 5D).

**Fig 6:**
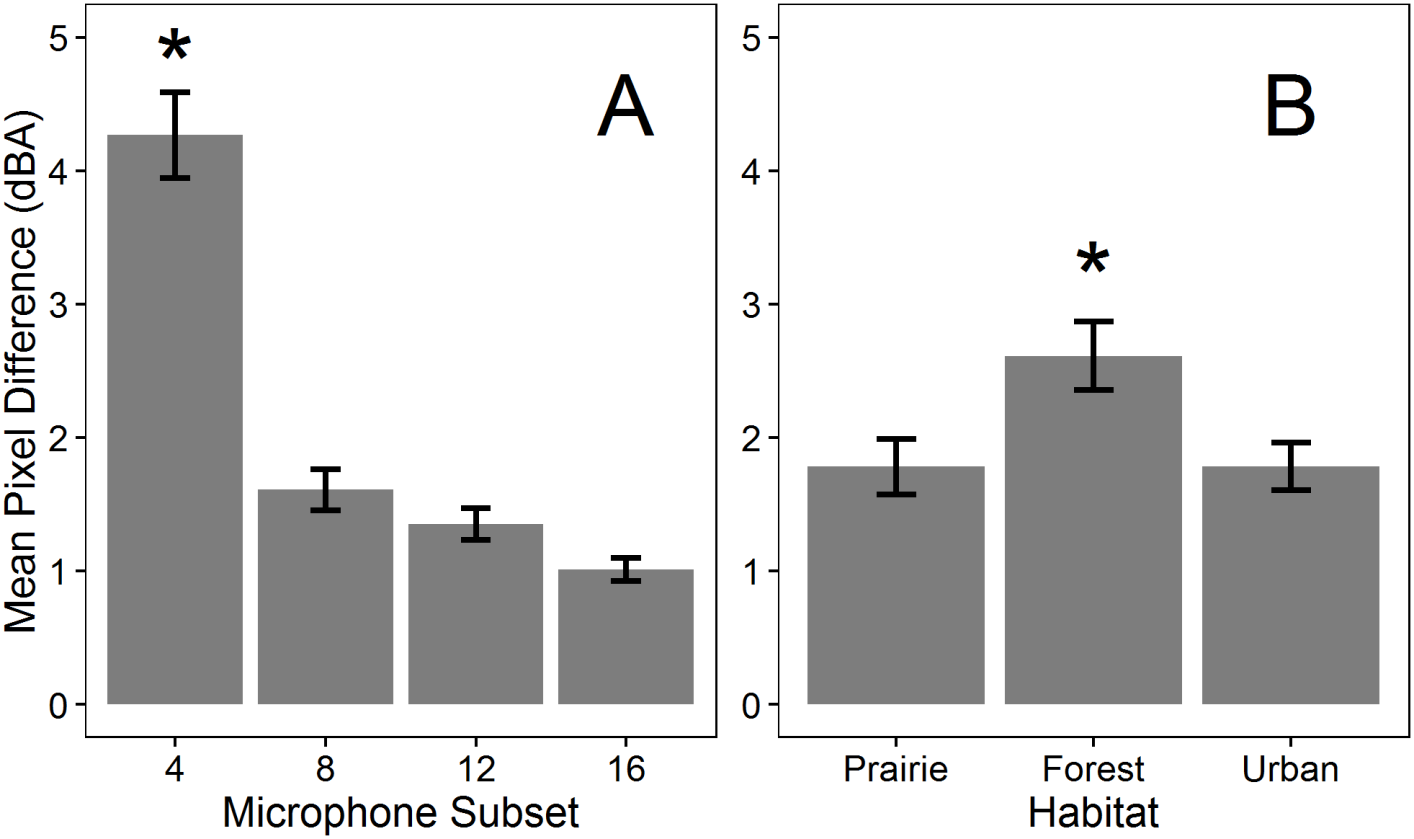
Map differences during noise introductions varied with microphone number (A) and across habitats (B). Mean pixel difference is the average difference in overall sound pressure levels (dBA) calculated by subtracting a subset map from the corresponding full array map and averaging overall all pixels. The asterisk denotes a significant difference as tested using Tukey HSD (P < 0.05). Error bars represent standard error.

With noise introductions, mean differences between full and subset maps were significantly different from zero (t > 3.79, P < 0.001; S3 Fig), although the value of the upper confidence interval is < 3 dBA for the 8-, 12- and 16-microphone subset maps (S2 Table). With noise, 4-microphone maps differed significantly more from full maps than the 8-, 12-, or 16-microphone maps (Table 2; Tukey HSD P < 0.05; Fig 6A); mean pixel differences between 4-microphone maps and full maps were greater than 4 dBA compared with differences of less than 2 dBA between full maps and the other configurations. Mean differences between 4-microphone maps and the others were larger than under ambient conditions (2.65, 2.92, and 3.26 dBA/pixel more, respectively), but still within the 3 dBA-difference threshold. Differences among the remaining subsets were not significant (Tukey HSD, P > 0.05).

**Table 2:** Results of a four-way ANOVA exploring influences on map differences during noise introductions.

	F_(d.f.)_	P
Habitat*Subset*Location*AdditionalMicrophones	1.17_(6,192)_	0.32
Habitat*Subset*Location	0.25_(6,192)_	0.96
Habitat*Subset*AdditionalMicrophones	0.67_(6,192)_	0.68
Habitat*Location*AdditionalMicrophones	1.075_(2,192)_	0.34
Subset*Location*AdditionalMicrophones	1.86_(3,192)_	0.14
Habitat*Subset	1.13_(6,192)_	0.35
Habitat*Location	0.36_(2,192)_	0.70
Habitat*AdditionalMicrophones	0.39_(2,192)_	0.68
Subset*Location	0.18_(3,192)_	0.91
Subset*AdditionalMicrophones	0.16_(3,192)_	0.93
Location*AdditionalMicrophones	32.35_(1,192)_	**< 0.001**
Habitat	11.25_(2,192)_	**< 0.001**
Subset	34.049_(3,192)_	**< 0.001**
Location	0.064_(1,192)_	0.80
AdditionalMicrophones	0.21_(1,192)_	0.65

The effect of noise introductions varied among habitats (Table 2, Fig 6B). Subset maps in forests differed more from their corresponding full maps than those in prairie and urban sites, although the mean pixel difference was small (0.83 dBA/pixel; Tukey HSD P < 0.05; Fig 6B). No interactions existed between habitat and the number of microphones within subsets (Table 2).

**Fig 7:**
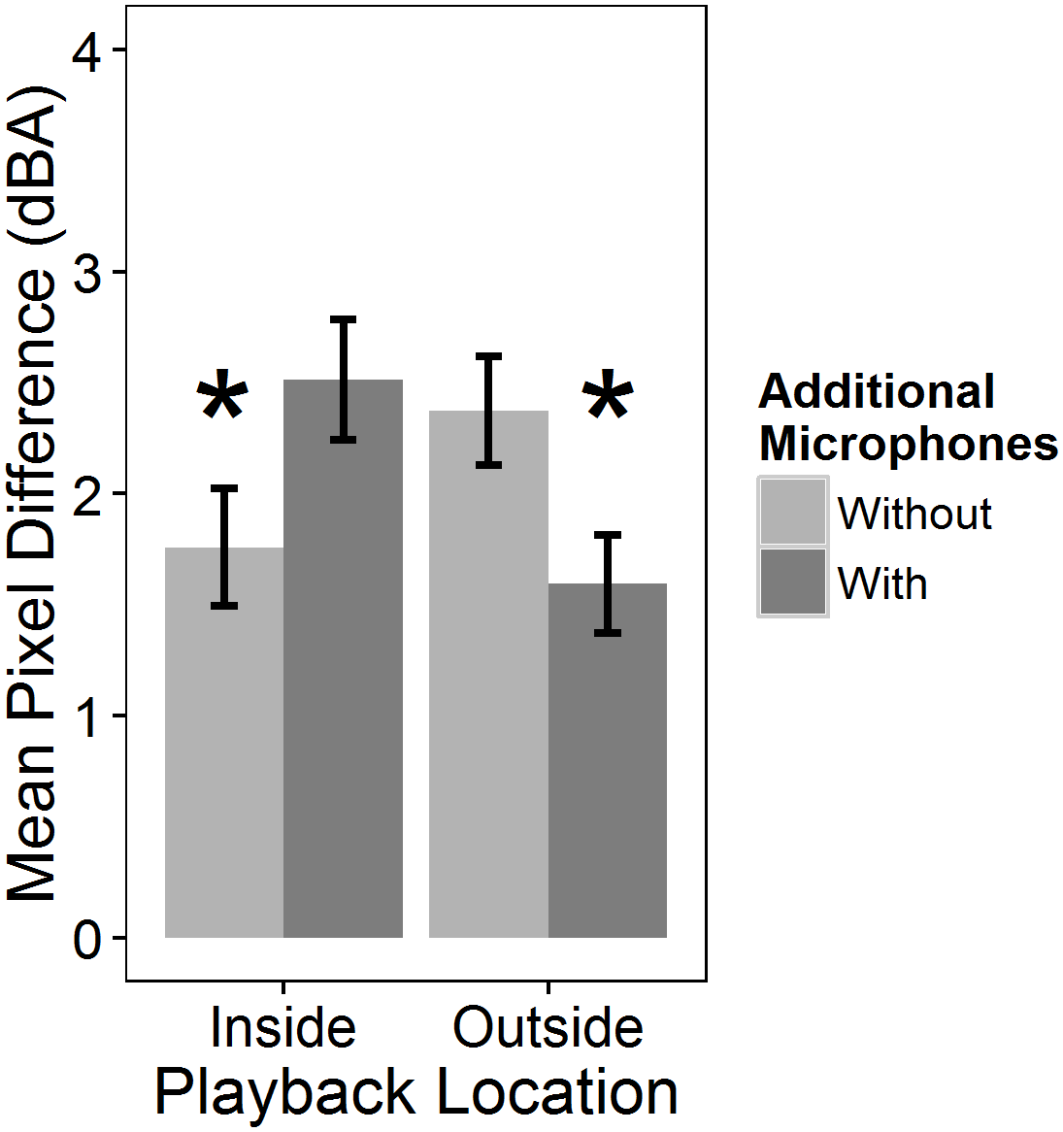
Effect of additional microphones on differences between maps depended on location of noise introductions. Mean pixel difference is the average difference in overall sound pressure levels (dBA) calculated by subtracting a subset map from the corresponding full array map and averaging overall all pixels. The asterisk denotes a significant difference as tested using Tukey HSD (P < 0.05). Error bars represent standard error.

A significant interaction existed between noise location and the use of additional microphones (Table 2). For noise introductions within arrays, differences between full and subset maps were greater with additional microphones around the noise source than without them (Tukey HSD P < 0.05), whereas additional microphones decreased differences between full and subset maps for introductions at array edges (Tukey HSD P < 0.05; Fig 7). Mean pixel differences were small (0.75 and 0.78 dBA/pixel for within and edge locations, respectively).

## Discussion

### Spatial variation in ambient sound on a small scale

The development of affordable autonomous recording systems has improved understanding of animal behavior and ecology (e.g., [30]), biodiversity (e.g., [41]) and the sound environment (e.g., [42]), including spatial variation in sound across landscape and continental scales (e.g., [21,23,24]). Here, we used microphone arrays to record the sound environment in three terrestrial habitats and mapped SPLs to explore variation in sound over small spatial scales. No site was homogeneous with respect to sound amplitude under ambient conditions and differences between maximum and minimum SPLs of up to 17 dBA occurred, revealing considerable spatial variation in SPLs over areas of only 60 m x 60 m (Fig 2). We suggest that single point measurements of SPLs are a first step in quantifying the sound environment, but additional sampling and sound mapping are needed to fully characterize acoustic habitats.

### How many microphones are needed to adequately map sound?

Large microphone arrays are costly to purchase and time-consuming to deploy and dismantle. Thus, we tested whether sequentially omitting microphones during the mapping process affected map results and we present recommendations based on these findings (Table 3). Under ambient conditions in each of three habitats, 4-microphone maps differed from full 24-microphone maps by less than 2 dBA/pixel, with smaller differences for the remaining subset maps (Fig 4A). Habitat type did not influence map differences under ambient conditions (Fig 4B), suggesting that similar approaches can be used across habitats. Thus, we propose that with only four microphones (equivalent to two autonomous recorders), and hence reduced cost and deployment time, the sound environment under ambient conditions can be recorded and mapped to visualize and understand spatial patterns in SPLs with a degree of accuracy adequate for most wildlife studies (e.g., [39]).

**Table 3:** Summary of findings and recommendations for sound mapping at small spatial scales (60 m x 60 m) under ambient conditions and with noise introductions in three habitats.

Sound condition	Differences between subset and full maps?	Does difference exceed 3-dBA threshold?	Influence of habitat on map differences?	Influence of location of noise source on map differences?	Recommendation
Ambient sound	Yes. 4-microphone maps differed more than other configurations from full maps.	No. Average map differences < 2 dBA.	No.	n/a	4-microphone array.
Noise source	Yes. 4-microphone maps differed more than other configurations from full maps.	Yes. 4-microphone maps differed by > 4 dBA from full maps. Other maps differed by < 3 dBA.	Yes. Forest maps differed from prairie and urban maps, but difference < 1 dBA.	Yes. Configuration of microphones should be adjusted depending on location of noise source.	Noise source within array: 8-microphone array. Noise source at edge of array: 4+4 microphone array.

The introduction of high-intensity noise significantly altered the sound environment and our conclusions about microphone number. Noise introductions increased overall SPLs (Table 1) and SPL gradients (> 40 dBA; S2 Fig), particularly in forest and prairie sites, resulting in greater spatial variation in SPLs (Fig 5) when noise was played. Unlike ambient conditions, 4-microphone maps differed by more than 4 dBA from full maps in noise (Fig 6A) and 4- microphone maps did not appear to reflect the location of the noise source. For example, the map for the 4-microphone array at the urban site (Fig 5A) showed a shift of the noise source from its true location within the array to the nearest microphone in the south-east corner of the map. These results suggest that the smallest array inadequately captured SPLs over space when noise was introduced.

However, eight microphones were sufficient. Maps made using input from eight or more microphones showed smaller differences (< 2 dBA/pixel) from full array maps than the four-microphone maps (Fig 6A). The location of the noise source mattered: during noise introductions at array edges, subset maps differed less from full maps with additional microphones (i.e. the 4+4 map) than without them, but the opposite occurred when introductions were placed within arrays (Figs 5 and 7). Location may have been influential because noise introductions within arrays were closer to microphones than introductions at the edges of arrays (mean ± SE distance of speaker to nearest microphone; within array = 4.20 ± 0.46 m; edge = 7.44 ± 0.70 m; t_(24,22)_ = -3.46, P < 0.001) and estimated values at the edge of maps created using IDW interpolation may be less accurate than those estimated at more central locations due to the lack of data receivers outside of the mapped area [43]. Mean differences were slight, but microphones around noise source(s) may be beneficial because their inclusion led to more accurate mapping of the location of noise on the edge of arrays. These findings suggest that with a single noise source, eight microphones captured the sound environment, but that microphone arrangement should vary depending on whether the noise sources was located within (8 microphone arrays) or on the edge (4+4 microphone arrays) of the sampling area (Table 3).

We mapped sound in three terrestrial habitats to evaluate whether different configurations were needed for different habitats. Under ambient conditions, urban sites experienced higher SPLs on average than forest or prairie sites (Table 1), but this difference did not affect mapping results, perhaps because SPL gradients were similar across habitats (S2 Fig, Fig 4A). With introduction of noise, forests had stronger SPL gradients than the other habitats (S2 Fig) and subset maps from forest sites showed larger differences from full maps than did those from prairie and urban sites (Fig 5A), even though we played noise at similar intensities across habitats. Forest maps may have differed from prairie and urban maps because of the greater potential for sound reflection and reverberation [44]. Habitats with many reflective surfaces, such as forests, experience considerable reverberation of sound waves [45]; microphones were positioned closely to reflective surfaces, i.e. leafed-out trees, therefore, microphones likely picked up energy from the playback as well as the energy in reflections, leading to higher SPLs at microphones near the noise source. Reverberation was limited in prairie sites because little vegetation occurred at the height of microphones and in urban sites because we avoided placing arrays near buildings and only scattered trees and bushes were present. Regardless, differences between subset and full maps in forests were still less than 3 dBA and no interactions were detected between habitat and the remaining factors (Table 2), suggesting that approaches to sound mapping may be generalizable across habitats.

We used a single stationary noise source, which mimicked noise emanating from building ventilation systems or gas compressors, for example. Adding microphones around the noise source, therefore, was simple. Noise sources may not be static and alternative approaches may be needed when noise sources move. Although not stationary per se, noise from cars and trucks is predictable in its location (i.e. roads and highways). Thus, a possible solution would be to position more microphones along roadways to capture traffic noise as it drives by; the optimal configuration and number of microphones along roads requires testing. Not all moving sources will be so predictable, however (e.g. recreational vehicle users could use different routes over time), and capturing noise from these sources likely requires a more complex configuration of microphones. In such circumstances, we speculate that more microphones within arrays would be needed, but further research is needed, including sound modeling and field validations, to understand how to produce accurate noise maps with moving noise sources.

The spatial extent of our study was small, but our approach can be extrapolated to larger areas. Under ambient conditions, 4-microphone arrays in 60 m x 60 m plots reasonably captured SPL variation. Using this basic layout, researchers could create grids over larger spatial extents while maintaining the relevant spacing with microphones placed at corners of 60m grids. Larger grids would capture the territories of many individuals in species with small territories (see below) or the territory or home range of small numbers of individuals if they range more widely. Under noise, additional microphones around noise sources may be required depending on their location and number within arrays.

### Mapping other sound metrics

We mapped overall A-weighted SPLs (dBA), but future studies could consider other measures of sound depending on the ecological question of interest [5,24,46]. Mapping unweighted SPLs (dB) provides insight into patterns of anthropogenic noise, as unweighted SPLs emphasize input from lower frequencies more than A-weighted measures used here [24]. Researchers can use software (e.g. MATLAB) to partition overall SPLs, that is, amplitude over all frequency bands, into octave or one-third octave frequency bands, which would allow them to focus on particular bands of interest. This would be particularly relevant to questions regarding noise impacts on animals, as mapping of low frequency bands would isolate contributions of noise from bands reflecting other sounds in the environment. Conversely, by mapping higher frequency bands that are more typical of birds or insects, questions regarding how animals respond spatially to con- and heterospecific signalers could be addressed. Mapping time-integrated SPL measurements could be used to identify areas that experience transient high-amplitude sound events (i.e. L_10_ or the SPL exceeded 10% of the time) as well as the SPLs characteristic of the environment (i.e. L_90_ or the SPL exceeded 90% of the time; [24]). Animals, including humans, respond differently to intermittent than chronic noise (e.g., [12,47]) and therefore mapping these values may also generate insight into how animals use space in relation to types of noise experienced. Finally, we focused primarily on mapping spatial variation in sound, but noise is not constant even over short time periods (S1 Movie) and it would be valuable to incorporate time to explore temporal variation in sound, possibly by mapping variability in SPL over time.

### Linking spatial variation in sound to animal ecology

The scale of our sound maps is relevant to diverse taxa. Many species of small mammals (e.g., [48]), birds (e.g., [49]), amphibians (e.g., [50]), lizards (e.g., [51]) and insects (e.g., [52]) have home ranges or territories as small or smaller than the area over which we mapped sound (60 m x 60 m). For these species, sound variation at small spatial scales could influence how organisms use space. In particular, given potentially negative effects of noise on animals (reviewed in [6,25]), organisms should pay attention to spatial variation in noise and avoid it where and when possible (e.g., [12,13,53,54]). For example, noise interferes with the ability of foraging animals to detect predators (e.g., [9]), therefore, if given the choice, we predict that organisms preferentially forage in quieter areas where detection probability may be higher or to adjust scanning frequency based on variation in noise. Noise also masks vocal signals and whereas we know that animals adjust signal structure and timing as a function of noise [6], we know little about whether they alter selection of signaling locations to minimize masking and optimize signal transmission. We predict that organisms select where to signal from within their territories and home ranges based on spatial variation in sound, avoiding noisy areas of their territories where masking will be more likely. Thus, organisms living in areas that vary spatially in sound could adjust signaling location, rather than or in addition to altering signal structure and timing, as a strategy to deal with noise. By considering spatial variation in noise we broaden consideration of the ways in which animals adjust behavior in noise.

In addition to new perspectives on individual behavior and space use, insight into population-level processes may be gained by knowing more about spatial variation in the sound environment. For example, densities of some bird populations are greater in urban than rural habitat (e.g., [55]). Could spatial variation in noise within urban habitats additionally influence the location of sites birds occupy (i.e. dispersion)? Moreover, if individuals avoid noise during habitat selection, we would expect competition for quiet areas and more aggressive individuals to be favored, particularly if reproductive success was lower in noisy areas. Noise negatively affects begging and provisioning of young by adult birds (e.g., [56]), which could have important demographic consequences for urban populations, but these effects could be tempered depending on the distribution of individuals in relation to noise. We suggest that a better understanding of spatial variation in sound when combined with individual- and population-level studies will generate new perspectives on the responses of animals to noise as well as sound more broadly (see also [5,17]).

To link sound mapping with animal ecology, sound maps can be developed in tandem with localization studies, behavioral observations, and radio-tracking, with the latter two approaches needed for study of less or non-vocal species. As localization studies also use microphone arrays, researchers interested in capturing data for both sound maps and localization should be aware that the array findings we set out here may need to be adjusted to improve localization of animals. The accuracy with which sound sources can be localized varies with signal characteristics, their location relative to microphone arrays, as well as the distance between microphones within arrays [31]. Thus, researchers interested in mapping both sound and animals in space need to explore different array layouts to achieve reliable results for both.

## Conclusion

Our goal was to understand spatial variation in sound pressure levels (SPLs) at small scales relevant to diverse animal species, as well as the number of microphones needed within arrays to accurately map the sound environment. We found considerable heterogeneity in SPLs over a small spatial scale under ambient sound conditions across habitats. Yet sound maps created using as few as four microphones showed only minor differences from maps generated using six times as many microphones and units. Therefore, we recommend that under ambient conditions four microphones may be placed at the corners of study plots to record the sound environment (Table 3). Under noisy conditions, more microphones were needed, but it remained that a relatively small number of microphones was sufficient: 8-microphone maps differed slightly from those using 28 microphones. For noise sources at edges of study plots, four of these microphones should surround the noise source; with noise within study plots, microphones can be distributed evenly within them. Future steps in the study of spatial variation in the sound environment include assessing drivers of sound variation across spatial scales and pairing studies of spatial variation in sound with those focused on habitat selection, space use, and behavior to advance understanding of the behavioral responses of animals to noise and sound more generally.

## Supporting Information

S1 Fig Spectrograms and power spectra of recordings used in noise introductions. Recording were made during rush hour (0700–0830) at intersections of major roads with average traffic flow of 19307.2 ± 2136.9 vehicles per 24-hour period (data from Kalamazoo Area Transportation Study, katsmpo.org).
(PDF)

S2 Fig Sound pressure level gradients across habitats and sound conditions. (PDF)

S3 Fig Sound maps illustrating overall sound pressure level differences between full and subset arrays during noise introductions. (PDF)

S1 Movie Animation of ambient sound pressure levels measured every 30 s over 5 min in each of three terrestrial habitats. (ZIP)

S1 Table Summary of under- and overestimation of mean pixel differences of full maps by subset maps across acoustic conditions. (DOCX)

S2 Table Results of analysis of mean pixel differences of subset maps relative to full maps. (PDF)
