# Supplementary material for: Uncovering Spatial Variation in Acoustic Environments Using Sound Mapping
Source: PLoS One. 2016 Jul 28;11(7):e0159883. doi: 10.1371/journal.pone.0159883 (PMC4965030; doi:10.1371/journal.pone.0159883)
Supplement: S1 Fig — Recording were made during rush hour (0700–0830) at intersections of major roads with average traffic flow of 19307.2 ± 2136.9 vehicles per 24-hour period (data from Kalamazoo Area Transportation Study, katsmpo.org). (PDF) [file pone.0159883.s001.pdf]

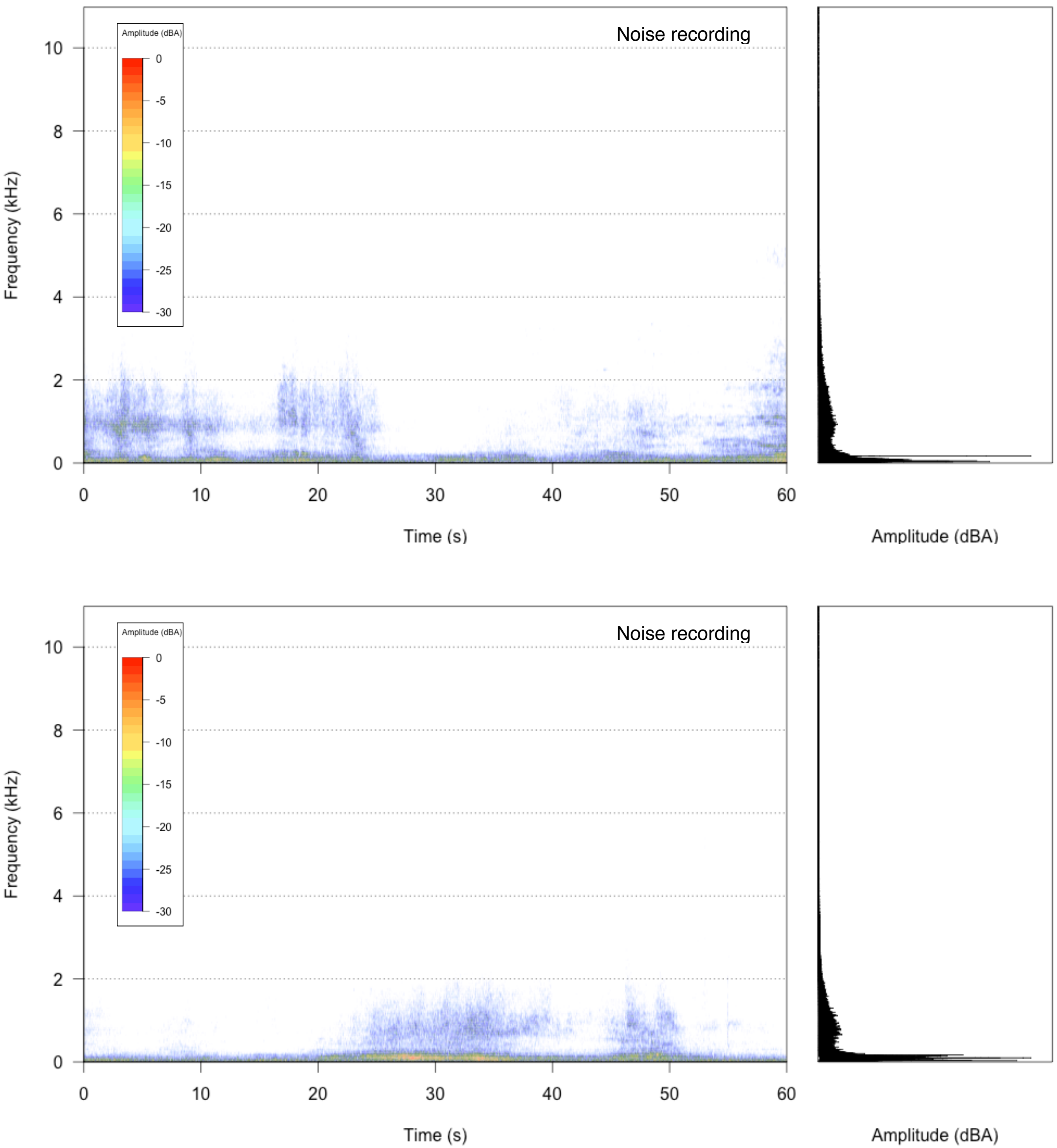

S1 Figure. Spectrograms (left) and power spectra (right) for five recordings of traffic noise used for noise introductions in the study "Sound mapping in terrestrial environments using microphone arrays". Recording were made during rush hour (0700-0830) at intersections of major roads with average traffic flow of 19307.2 ± 2136.9 vehicles per 24-hour period (data from Kalamazoo Area Transportation Study, katsmpo.org). Each recording was 5-min in duration; we randomly sampled 1-min of the recording to illustrate frequency and amplitude characteristics of noise recordings. Recordings were similar with most energy within the frequency range of 0-2 kHz. Spectrograms and power spectra were generated using the package seewave in R v. 3.1.1 (R Core Team 2014).

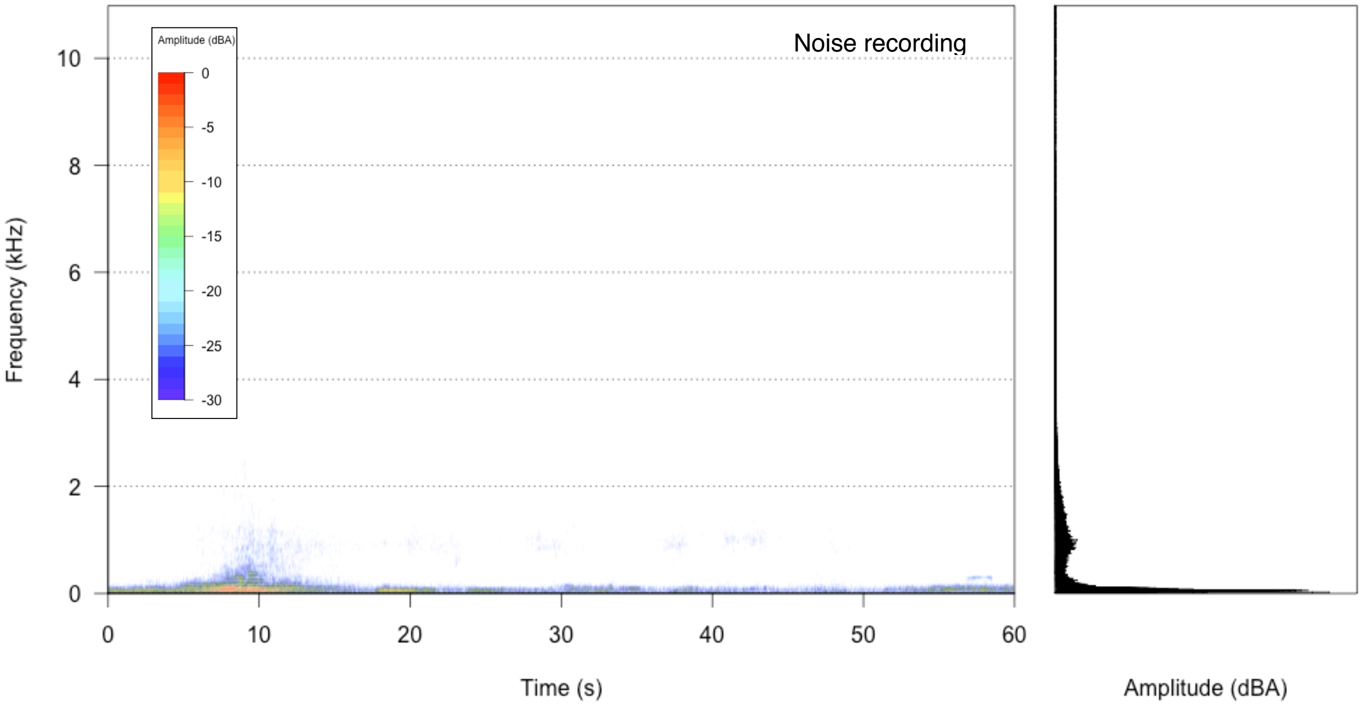

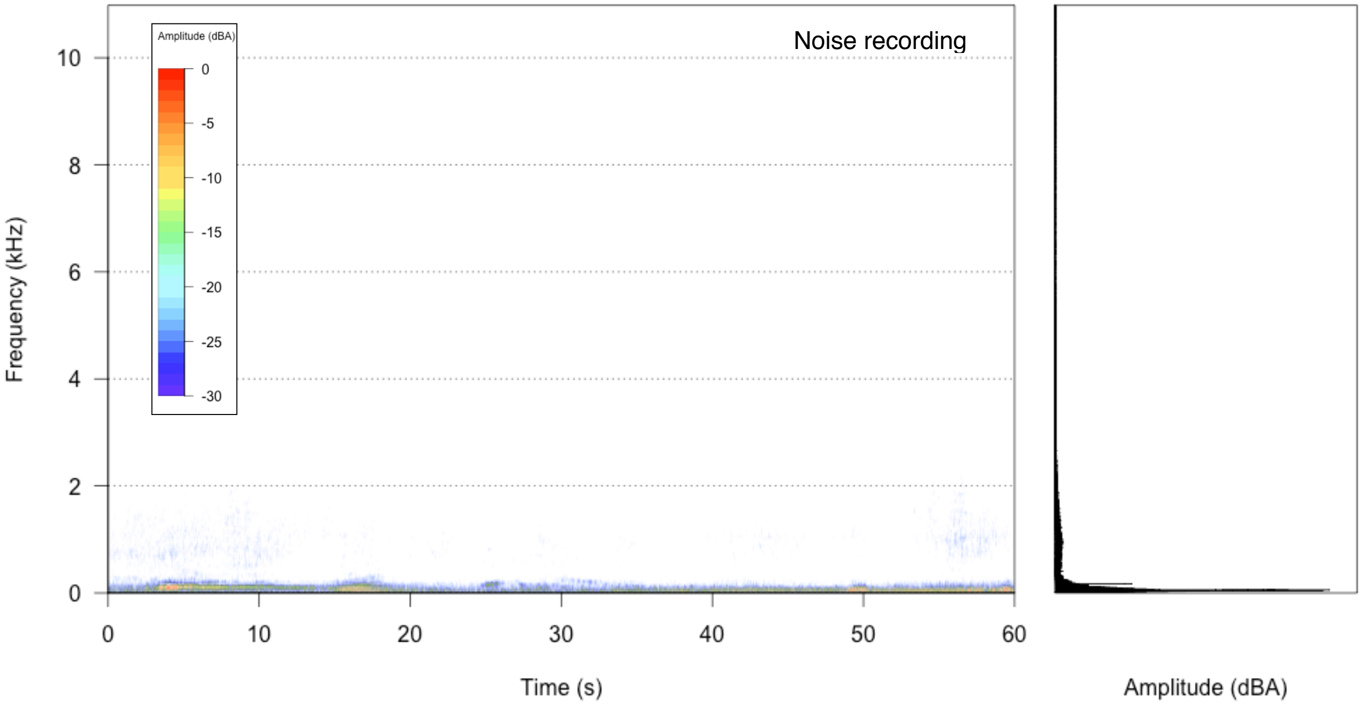

S1 Figure continued.

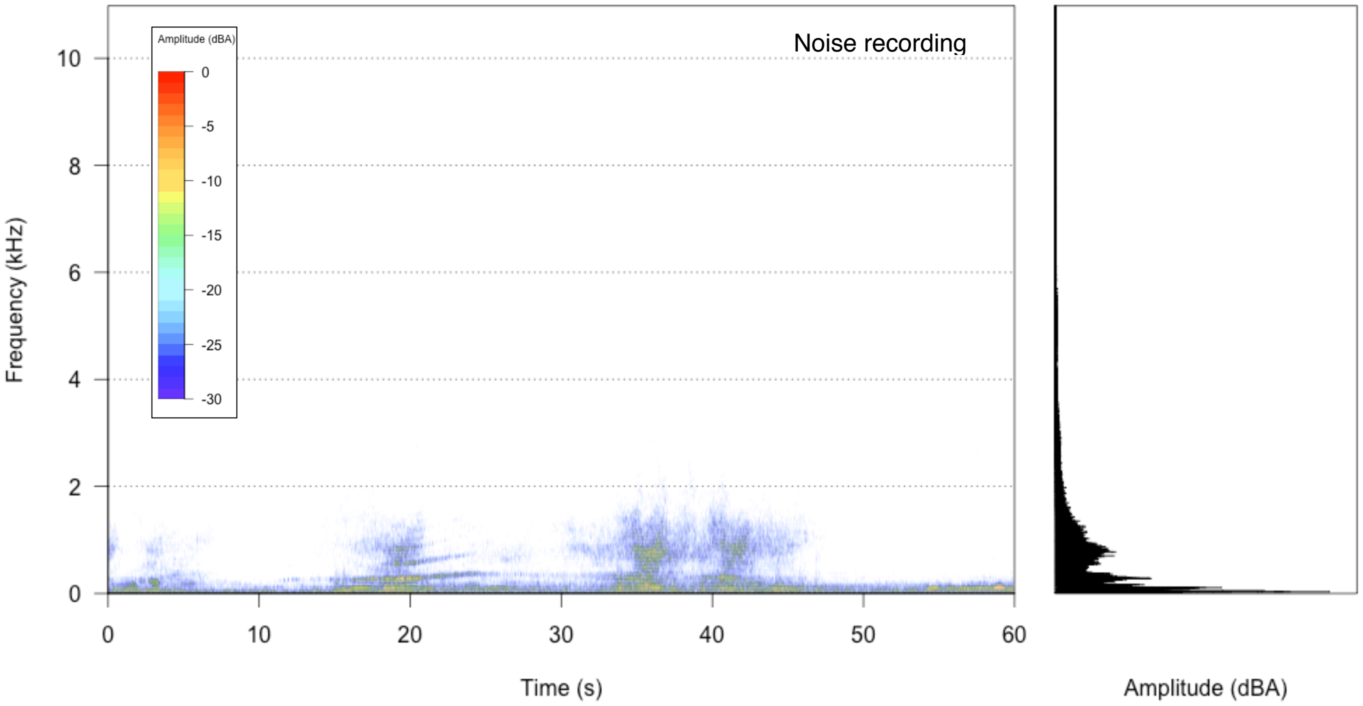

S1 Figure continued.
