# Supplementary material for: Uncovering Spatial Variation in Acoustic Environments Using Sound Mapping
Source: PLoS One. 2016 Jul 28;11(7):e0159883. doi: 10.1371/journal.pone.0159883 (PMC4965030; doi:10.1371/journal.pone.0159883)
Supplement: S2 Fig — (PDF) [file pone.0159883.s002.pdf]

S2 Fig. Sound pressure level gradients across habitats and sound conditions.

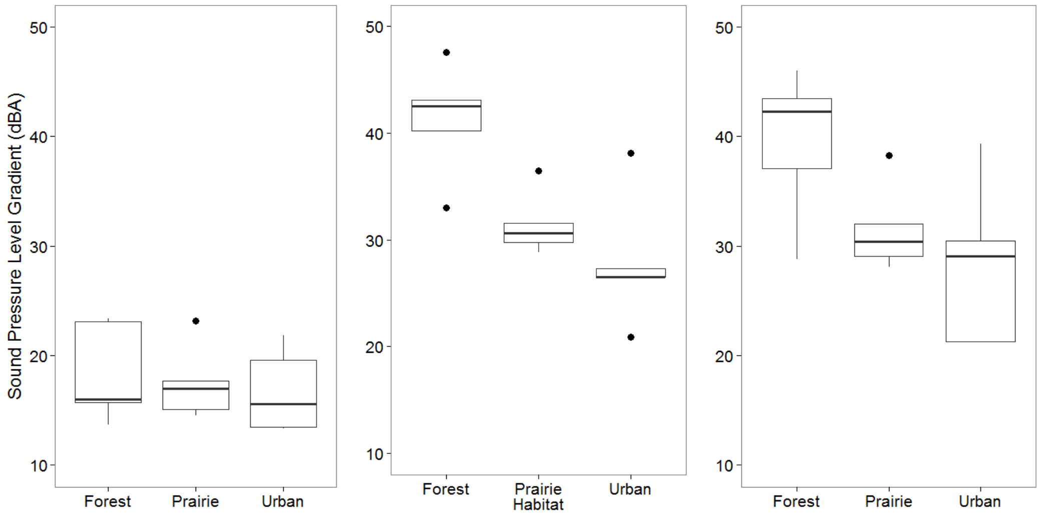

Sound pressure level (SPL) gradients (dBA) were similar across habitats under (A) ambient conditions ($F_{2,12}$ =0.21, P = 0.8); however, during noise introductions, differences between maximum and minimum SPLs (A) within arrays and (B) at the edge of arrays differed significantly across habitats. SPL gradients were greater in forest arrays than prairie or urban when noise was introduced within arrays ($F_{2,12}$ = 9.41, P = 0.004) and greater than urban arrays when noise was introduced at the edge ($F_{2,12}$ = 4.21, P = 0.041).
