# Supplementary material for: Uncovering Spatial Variation in Acoustic Environments Using Sound Mapping
Source: PLoS One. 2016 Jul 28;11(7):e0159883. doi: 10.1371/journal.pone.0159883 (PMC4965030; doi:10.1371/journal.pone.0159883)
Supplement: S3 Fig — (PDF) [file pone.0159883.s003.pdf]

S3 Fig. Sound maps of noise introductions illustrating overall sound pressure level differences between full and subset arrays.

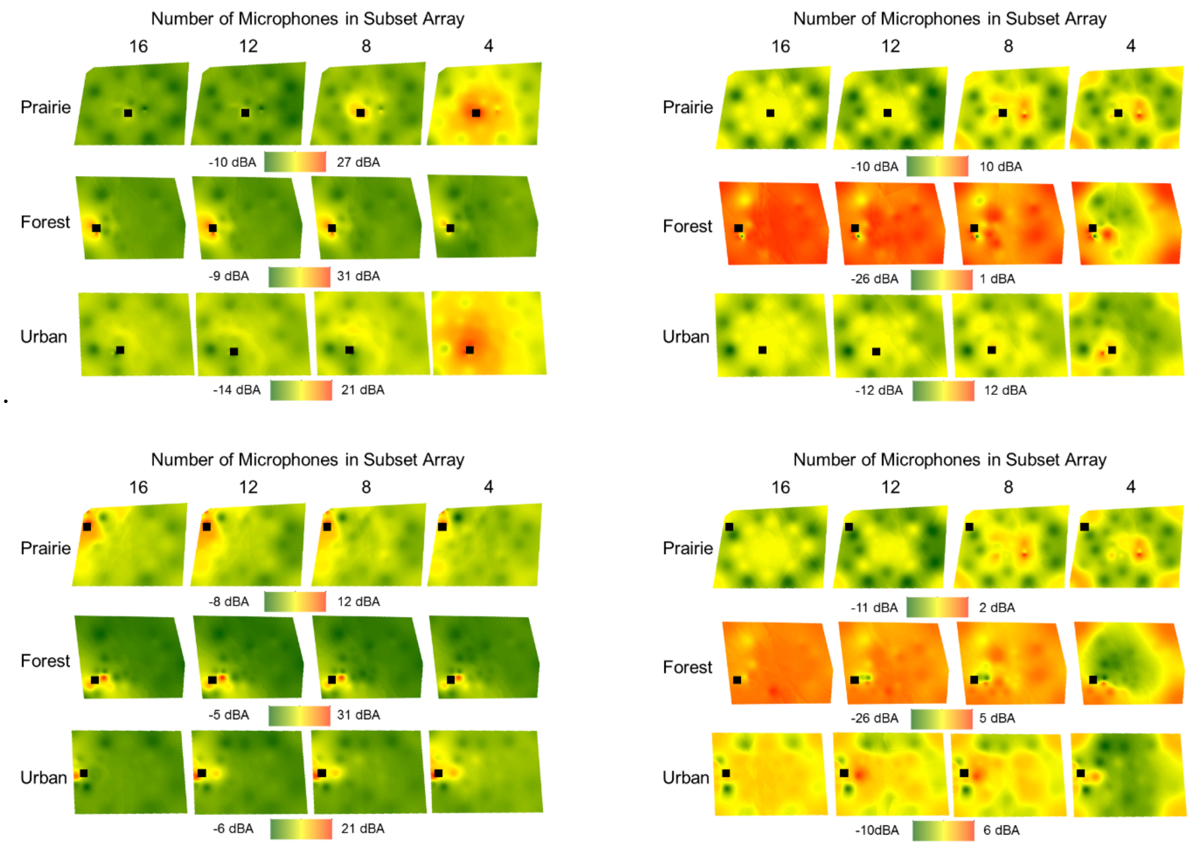

Examples sound maps in three habitats illustrating differences in overall SPLs (dBA) between full array maps (24+4) and subset array maps during noise introduction (■) within arrays without (A) and with (B) additional microphones around noise source, and noise introduction at the edge of arrays without (C) and with (D) additional microphones around the noise source. Within habitat, maps in (A) and (B) illustrate the same randomly selected point in time, as do (C) and (D), which are also the same points in time and study sites as those depicted in Fig. 5. Note that the ranges and values of sound pressure levels (dBA) differ among maps for the three habitats.
