# Supplementary material for: Uncovering Spatial Variation in Acoustic Environments Using Sound Mapping
Source: PLoS One. 2016 Jul 28;11(7):e0159883. doi: 10.1371/journal.pone.0159883 (PMC4965030; doi:10.1371/journal.pone.0159883)
Supplement: S1 Table — (DOCX) [file pone.0159883.s005.docx]

S1 Table. Summary of under- and overestimation of mean pixel differences of full maps by subset maps across acoustic conditions. Habitats are combined. Data are the number of subset maps that under- or overestimate full maps and the mean value of the differences between full and subset maps (dBA).

|  |  | Ambient conditions | | Noise introductions | | | | | | | |
| --- | --- | --- | --- | --- | --- | --- | --- | --- | --- | --- | --- |
|  |  |  |  | Inside array | | | | Edge of array | | | |
| Number of microphones |  |  |  | Without additional microphones | | With additional microphones | | Without additional microphones | | With additional microphones | |
|  |  | Under | Over | Under | Over | Under | Over | Under | Over | Under | Over |
| 4 | Number | 6 | 9 | 15 | 0 | 0 | 15 | 10 | 5 | 0 | 15 |
|  | Mean | 0.77 | -1.34 | 4.85 | n/a | n/a | -4.91 | 3.95 | -1.55 | n/a | - 5.32 |
| 8 | Number | 3 | 12 | 11 | 4 | 1 | 14 | 11 | 4 | 0 | 15 |
|  | Mean | 0.44 | -0.52 | 1.18 | -0.23 | 0.39 | -2.73 | 1.19 | -0.90 | n/a | -2.30 |
| 12 | Number | 2 | 13 | 9 | 6 | 0 | 15 | 9 | 6 | 0 | 15 |
|  | Mean | 0.58 | -0.35 | 1.16 | -1.00 | n/a | -1.99 | 1.14 | -0.64 | n/a | -1.61 |
| 16 | Number | 4 | 11 | 8 | 7 | 0 | 15 | 11 | 4 | 0 | 15 |
|  | Mean | 0.42 | -0.26 | 1.47 | -0.56 | n/a | -1.23 | 0.85 | - 0.86 | n/a | -0.98 |
