# Supplementary material for: Uncovering Spatial Variation in Acoustic Environments Using Sound Mapping
Source: PLoS One. 2016 Jul 28;11(7):e0159883. doi: 10.1371/journal.pone.0159883 (PMC4965030; doi:10.1371/journal.pone.0159883)
Supplement: S2 Table — (PDF) [file pone.0159883.s006.pdf]

S2 Table. Results of analysis of mean pixel differences of subset maps relative to full maps.

Mean pixel differences from full arrays (Ambient: 24; Noise Introductions: 24+4) were greater than zero for all subsets under ambient conditions and with noise introductions (all t-tests P <0.001). However, the value of the upper confidence interval for mean pixel differences was less than 1 dBA for 8-, 12- and 16-microphone subset maps under ambient conditions and less than 3 dBA with introduced noise. These results suggest that the differences in full array maps and 8-, 12- and 16-microphone maps were slight. 'Within' and 'Edge' indicate the location of noise introductions within arrays. 'No. Add. Mics.' and 'Add. Mics.' indicate the presence or absence of additional microphones around speaker.

	Subset Array											
	4 Microphones			8 Microphones			12 Microphones			16 Microphones		
		95% CI			95% CI			95% CI			95% CI	
Sound Condition	$t_{(d.f.)}$	U	L	$t_{(d.f.)}$	U	L	$t_{(d.f.)}$	U	L	$t_{(d.f.)}$	U	L
Ambient	$5.98_{(14)}$	2.066	0.96	$5.95_{(14)}$	0.73	0.34	$5.39_{(14)}$	0.50	0.21	$4.89_{(14)}$	0.41	0.16
Noise introduction												
Within; No Add. Mics.	$5.085_{(14)}$	5.49	2.23	$3.79_{(14)}$	1.70	0.47	$4.28_{(14)}$	1.64	0.55	$5.55_{(14)}$	1.36	0.60
Within; Add. Mics.	$7.62_{(14)}$	6.38	3.57	$7.58_{(14)}$	2.87	1.61	$6.63_{(14)}$	2.27	1.16	$6.45_{(14)}$	1.49	0.75
Edge; No Add. Mics.	$7.24_{(14)}$	5.84	3.17	$7.81_{(14)}$	2.74	1.56	$8.04_{(14)}$	2.13	1.23	$6.20_{(14)}$	1.57	0.76
Edge; Add. Mics.	$7.022_{(14)}$	4.87	2.59	$4.27_{(14)}$	1.45	0.48	$5.082_{(14)}$	1.29	0.53	$4.63_{(14)}$	1.13	0.41
